# The relationship of genetic risk score with cardiometabolic risk factors: a cross-sectional study

**DOI:** 10.1186/s12872-022-02888-z

**Published:** 2022-11-02

**Authors:** Fatemeh Gholami, Niloufar Rasaei, Mahsa Samadi, Mir Saeid Yekaninejad, Seyed Ali Keshavarz, Gholamali Javdan, Zahra Karimi, Khadijeh Mirzaei

**Affiliations:** 1 Department of Community Nutrition, School of Nutritional Sciences and Dietetics, Tehran University of Medical Sciences (TUMS), P. O Box 6446, 14155 Tehran, Iran; 2 Department of Epidemiology and Biostatistics, School of Public Health, Tehran University of Medical Science, Tehran, Iran; 3 Department of Clinical Nutrition, School of Nutritional Sciences and Dietetics, Tehran University of Medical Sciences, Tehran, Iran; 4 Food Health Research Center, Hormozgan University of Medical Sciences, Bandar Abbas, Iran; 5 Food Microbiology Research Center, Tehran University of Medical Sciences, Tehran, Iran

**Keywords:** Cardiometabolic risk factors, Genetic risk score, Obesity

## Abstract

**Background & aims:**

For more than eight decades, cardiovascular disease (CVD) has remained the leading cause of death in the world. CVD risk factors are multifaceted, with genetics and lifestyle both playing a role. The aim of this study was to investigate the association between a genetic profile risk score for obesity GRS and cardio-metabolic risk factors in overweight and obese women.

**Methods:**

The current cross-sectional study was conducted on 391 overweight and obese women. The genetic risk score was created by combining three single nucleotide polymorphisms [MC4R (rs17782313), CAV-1 (rs3807992), and Cry-1 (rs2287161)]. Anthropometric measurements, blood pressure, and some blood parameters were measured by standard protocols.

**Results:**

A significant association between the GRS and some of cardiometabolic risk factors variables such as body mass index (β = 0. 49, 95%CI = 0.22 to 0.76, p < 0.001), waist circumference (β = 0. 86, 95%CI = 0.18 to 1.54, p = 0.01), body fat mass (β = 0. 82, 95%CI = 0.25 to 1.39, p = 0.005), %body fat (β = 0. 44, 95%CI = 0.06 to 0.82, p = 0.02), and hs-CRP (β = 0.46, 95% CI = 0.14 to 0.78, p = 0.005) was observed in crude model. After adjustment for confounding factors (age, BMI, and physical activity), a significant positive association was observed between BMI (p = 0.004), WC (p = 0.02), body fat mass (p = 0.01), %BF (p = 0.01), hs-CRP (p = 0.009), and GRS. In addition, we discovered a significant negative association between the GRS and BMC (= -0.02, 95%CI = -0.05 to -0.001, p = 0.04). But other variables did not show any significant association with GRS among obese and overweight women.

**Conclusion:**

We found a significant positive association between GRS, including MC4R (rs17782313), CAV-1 (rs3807992), and Cry-1 (rs2287161) and cardiometabolic risk factors among overweight and obese Iranian women.

## Introduction

Cardiovascular disease (CVD) is one of the major contributors to mortality and the global burden of disease [1, 2], accounting for one-third of all deaths worldwide [3]. Approximately 17.8 million deaths in 2017 were attributable to CVDs [2], and the number is expected to increase to 23.6 million by 2030 [4], with a similar rising mortality rate in Iran [5]. The risk factors of CVD are multifactorial, including dyslipidemia, high blood pressure, insulin resistance, inflammation, and high BMI and waist circumference (WC) [6–9]. Furthermore, genetic predisposition has been identified as a significant predictor of CVDs.

Large-scale genome-wide association studies (GWAS) have identified several novel genetic variants correlated with CVD and obesity [10–12]. Moreover, the associations between genetic factors and CVD could be easily determined by using GRS, calculated through the accumulation of risk alleles for each single nucleotide polymorphism (SNP) in samples much smaller than those required for GWAS [11, 13, 14]. MC4R (melanocortin 4 receptor) rs17782313 variant has been reported to have an association with higher BMI in children and adults [15–18]. Furthermore, the risk allele C for MC4R rs17782313 was linked to cardiovascular risk factors such as insulin resistance and hypertension, as well as a higher susceptibility to obesity and inflammation [19–23]. Caveolin-1 (CAV-1) is a major structural protein of caveolae, [24] which is abundant in adipocytes [25]. Over the preceding decade, a large variety of studies have implicated CAV-1 SNP in the development of hypertension, dyslipidemia, and atherosclerosis [26–28]. It is worth noting that A-allele carriers of the CAV-1 rs3807992 had significantly higher BMI [29, 30]. Cryptochromes (Cr*y*) 1, the principal component of the negative limb of the core clock, appears to play important roles in metabolism [31] such as regulating glucose homeostasis [32]. Thus, it could contribute to the risk of insulin resistance [33]. C-allele of the Cry-1 rs2287161 polymorphism, in turn, contributes to the significant greater BMI [34]. So, the aforementioned genetic variants were previously reported to be individually associated with overweight/obesity in some populations [35, 36].

Given the foregoing, GRS, as a result of the combined impact of multiple SNPs, is thought to be a useful tool for predicting cardiometabolic risk factors and increasing the power to detect them. Furthermore, no evidence has been generated up to now on the association between cardio-metabolic traits and obesity-GRS, based on the aforementioned genetic factors. Therefore, the present study has been undertaken to compute the GRS through traits associated with obesity-related genetic markers, namely, MC4R (rs17782313), CAV-1(rs3807992), and Cry-1 (rs2287161), to improve the identification of overweight and obese women at a higher risk of developing cardio-metabolic risk factors.

## Methods and materials

### Study population

This cross-sectional study involved 391 overweight or obese women. The age range of the participants was 18 to 68 years, and the BMI range of the participants was 25–40 kg/m2, which refers to health centers in Tehran, Iran. Participants had no common ancestry and were not related. The following conditions were excluded: type I and type II diabetes, cardiovascular disease, malignancies, kidney disease, thyroid disease, menopause, pregnancy, lactation, smoking, any acute or chronic diseases, weight loss supplements, particular diets during the last year, and taking thyroid, lipid, glucose, or blood pressure drugs. All participants signed written informed consents before enrollment in the study, which was reviewed and approved by the Tehran University of Medical Sciences (TUMS) in Tehran, Iran. Ethical approval, and associated number IR.TUMS.MEDICINE.REC.1400.1515, was obtained from The ethics Commission of the Tehran University of Medical Sciences. All research was performed in accordance with relevant guidelines and regulations.

### Body composition analysis

By strictly following the techniques, procedures, and precautions outlined in the manufacturer’s protocol, we measured body composition using a bioelectrical impedance analyzer (BIA), InBody 770 scanner (Inbody Co., Seoul, Korea) protocol [37]. The instructions specified by the manufacturer require participants to remove extra clothing and metal utensils, such as earrings, rings, and watches, as well as removable shoes, coats, and sweaters. The following body composition measurements are taken: fat mass (FM), fat-free mass (FFM), skeletal muscle mass (SMM), waist circumference (WC), waist to hip ratio (W.H.R), and others.

### Anthropometric indices

Using a non-stretch tape measure, we measured and recorded participants’ heights, standing up and unshod, with a precision of 0.5 cm. For hip and waist circumference, the most prominent part and the narrowest part, respectively, were marked and measured with a precision of 0.5 cm. Various anthropometric characteristics, such as weight and BMI, were determined by BIA.

### Physical activity assessment

All participants were assessed on their physical activity during the last week using the short form of the International Physical Activity Questionnaire (IPAQ). Physical activity in the last week was measured using IPAQ, a validated self-report instrument [38].

### Biochemical and hormonal determination

Venous blood was collected between 8:00 a.m. and 10:00 a.m. following an overnight fast. The serum was centrifuged, aliquoted, and stored at − 80 °C, and all samples were analyzed by using a single assay technique. Glucose oxidase-phenol 4-aminoantipyrine peroxidase (GOD-PAP) was used to measure fasting blood glucose (FBS). A glycerol-3-phosphate oxidase–phenol 4-aminoantipyrine peroxidase (GPOPAP) enzymatic endpoint was used to measure triglyceride (TG) and total cholesterol (TC). A direct enzymatic clearance assay was used to measure low-density-lipoprotein (LDL), and high-density lipoprotein (HDL) cholesterol. MCP-1, hs-CRP, and galectin were measured via standard protocols. Plasminogen activator inhibitor-1 (PAI-1) (Human PAI-1*96 T ELISA kit Crystal Company) was measured in triplicate. An immunoturbidimetric assay (high sensitivity assay, Hitachi 902) was used to measure serum inflammatory markers. The data on insulin minimum detectable concentration was 1.76 mIU/mL and the intra CV was 2.19%, and inter CV was 4.4%. Homeostasis model assessment (HOMA), a measure of HOMA-IR, was calculated as [(fasting plasma glucose × fasting serum insulin)/22.5] [39]. Randox Laboratories (Hitachi 902) kit was used for all measurements. All samples were assessed by standard methods at the Nutrition and Biochemistry Laboratory of the School of Nutritional and Dietetics at TUMS.

### Genotyping and GRS

The DNA was extracted using the salting out method [40]. Subsequently, the DNA integrity was observed using a 1% agarose gel, whereas DNA concentration was quantified using a Nanodrop 8000 Spectrophotometer (Thermo Scientific, Waltham, MA, USA). The PCR-allele technique performed by the TaqMan Open Array (Life Technologies Corporation, Carlsbad, CA, USA), was used for genotyping of the SNPs [41]. The MC4R gene primer was selected based on a previous study [42]. For MC4R (rs17782313), we used polymerase chain reaction (PCR) with the following primers: forward primer 5- AAGTTCTACCTACCATGTTCTTGG-3 and reverse primer 5-TTCCCCCTGAAGCTTTTCTTGTCATTTTGAT-3. Then, fragments containing three genotypes were distinguished: CC, CT, and TT. We used PCR with the following primers for CAV-1 (rs3807992): forward primer 3′AGTATTGACCTGATTTGCCATG 5′ and reverse primer 5′ GTCTTCTGGAAAAAGCACATGA 3′. Then, fragments containing three genotypes were distinguished: GG, GA, and AA. For Cry1 (rs2287161), we used PCR following primers: forward primer 5′-GGAACAGTGATTGGCTCTATCT − 3′ and reverse primer 5′-GGTCCTCGGTCTCAAGAAG-3′. Then, fragments containing three genotypes were distinguished: CC, GC, and GG.

The GRS was created by combining three single nucleotide polymorphisms [MC4R (rs17782313), CAV-1 (rs3807992), and Cry-1 (rs2287161)] that had previously been linked to obesity-related traits in GWAS and other studies [17, 35, 43–45]. Each SNP was recoded as 0, 1, or 2 according to the number of risk alleles for higher BMI. Following that, the unweighted GRS was calculated by adding the number of risk alleles from the three SNPs. The GRS scale ranges from 0 to 6, with each point corresponding to one risk allel. Higher scores indicate a greater genetic susceptibility to higher BMI or body weight [46].

### Statistical analyses

Kolmogorov-Smirnov test was used to check the normal distribution of data. Descriptive analysis was used to assay the general characteristics of participants by the mean ± standard deviation, minimum and maximum. Analysis of variance (ANOVA) and analysis of covariance (ANCOVA) were performed to compare the body composition, blood pressure, the metabolic and inflammatory profiles between subjects. Linear regression was used in the crude model and adjusted models to evaluate the associations of cardiometabolic risk factors (dependent variable) and GRS (independent variable). Adjustments were made for age, physical activity, and BMI. All statistical analysis was performed by using the SPSS version 23.0 (SPSS, Chicago, IL, USA). All reported P-values were two-sided, and a P-value lower than 0.05 was considered statistically significant.

Finally, we draw a forest plot for linear regression coefficients for a better vision of associations between GRS and each of the cardiometabolic variables, and also for comparing the crude and adjusted models. For this purpose, we utilized the “forestplot” package (version 2.0.1) in “R Programming” software (version 4.0.3).

## Result

### Study population characteristics

This cross-sectional study was conducted on 391 overweight or obese but apparently healthy women. The means and standard deviation (SD) of age, weight, and BMI of individuals were 36.65 ± 9.08 years, 80.75 ± 11.52 kg, and 31.03 ± 3.87 kg/m2, respectively. The general characteristics of study participants are given in more detail in Table 1.

**Table 1:** Characteristics of the investigating subjects

Variable	Mean	SD	Minimum	Maximum
Age (years)	36.65	9.08	18.00	64.00
Body weight (Kg)	80.75	11.52	59.50	122.40
BMI (Kg/m^2^)	31.03	3.87	25.00	40.70
**Body composition**				
WC (cm)	99.22	9.60	79.60	131.30
WHR (ratio)	0.93	0.05	0.81	1.13
BFM (kg)	34.28	8.04	19.40	66.00
BF (%)	42.04	5.31	15.00	56.20
BMR (kcal)	1368.65	118.68	1092.00	1833.00
BMC (g)	2.65	0.34	1.89	3.93
SMM (kg)	25.48	3.37	17.30	37.90
**Blood pressure**				
SBP (mmHg)	111.32	13.44	76.00	159.00
DBP (mmHg)	77.65	9.55	51.00	111.00
**Blood parameters**				
FBS (mg/dl)	87.26	9.68	67.00	137.00
Total cholesterol (g/dl)	183.92	35.32	104.00	344.00
TG (mg/dl)	120.80	68.67	37.00	512.00
HDL (mg/dl)	46.45	10.63	18.00	82.00
LDL (mg/dl)	94.56	23.79	34.00	156.00
hs-CRP (mg/L)	5.08	4.54	0.00	22.73
HOMA index	3.35	1.27	1.29	9.19
Insulin (mIU/ ml)	1.21	0.23	0.60	1.99
MCP-1 (ng/ml)	50.57	92.91	0.40	575.40
Galectin3 (ng/ml)	4.10	7.30	0.15	32.29
PAI-1 (ng/ml)	16.10	29.92	0.52	202.00

SD: Standard deviation; BMI: Body mass index; WC: waist circumference; WHR: waist height ratio; BFM: body fat mass; BF: body fat; BMR: Basal metabolic rate; BMC: bone mineral content; SMM: skeletal muscle mass; SBP: Systolic blood pressure; DBP: Diastolic Blood Pressure; FBS: fasting blood sugar; TG: Triglyceride; LDL: Low density lipoprotein; HDL: High density lipoprotein; hs-CRP: High-sensitivity C-reactive protein; MCP-1: monocyte chemoattractant protein; PAI-1: Plasminogen Activator Inhibitor 1

### Difference in means of cardiometabolic variables across GRS

**Fig. 1:**
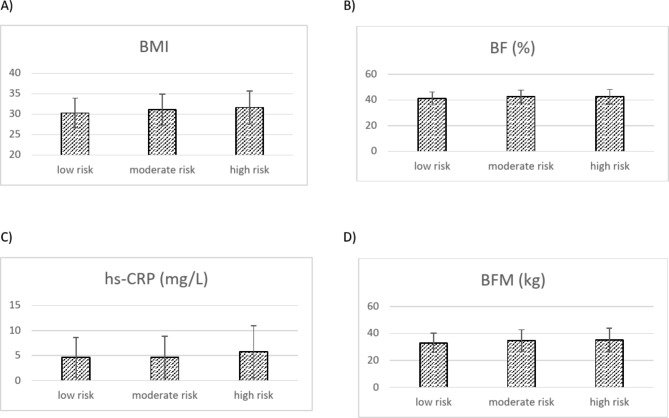
Difference in means of cardiometabolic variables across GRS. BMI: Body mass index;BFM: body fat mass; BF: body fat; hs-CRP: High-sensitivity C-reactive protein

A total of 391 Iranian women were categorized based on their genetic risk score. The genetic risk score was divided into low risk, moderate risk, and high risk of genetic risk score and study variables were reported and compared across the genetic risk score. After categorization, we found that women at high risk of GRS had a significantly higher BMI (p = 0.01). The results also displayed a borderline significant difference across the genetic risk scores for body fat mass (p = 0.05), percentage of body fat (p = 0.05), serum HDL concentration (p = 0.08), and CRP (p = 0.06). After adjustment for confounding factors (BMI, age, and physical activity), BMI (p = 0.04) and percentage of body fat (p = 0.03) maintained their significant differences. Also, a borderline significant difference was observed for body fat mass (p = 0.05) and CRP (p = 0.05) in the adjusted model (Table 2) (Fig. 1).

**Table 2:** Mean and SD of anthropometric body composition, blood parameters and blood pressure across to GRS.

Variables†	GRS				
	**Low risk (164)**	**Moderate risk (97)**	**High risk (130)**	**P-value**	**P-value**_**b**_
	> 3	3	3 <		
Age (years)	36.66 ± 9.51	37.20 ± 8.85	36.27 ± 8.83	0.72	0.76
Body weight (Kg)	79.88 ± 10.29	80.50 ± 11.56	81.73 ± 12.56	0.38	0.69^a^
BMI (Kg/m^2^)	30.30 ± 3.60	31.16 ± 3.79	31.63 ± 4.07	**0.01**	**0.04**
**Body composition**					
WC (cm)	98.05 ± 9.01	99.41 ± 9.48	100.15 ± 10.16	0.18	0.25 ^a^
WHR (ratio)	0.93 ± 0.05	0.93 ± 0.04	1.55 ± 7.51	0.43	0.50 ^a^
BFM (kg)	32.98 ± 7.22	34.75 ± 7.96	35.21 ± 8.70	**0.05**	**0.05** ^a^
BF (%)	41.18 ± 5.10	42.61 ± 4.95	42.46 ± 5.67	**0.05**	**0.03** ^a^
BMR (kcal)	1377.30 ± 115.06	1376.30 ± 164.83	1371.17 ± 123.62	0.91	0.58 ^a^
BMC (g)	2.68 ± 0.33	2.64 ± 0.35	2.63 ± 0.35	0.37	0.21 ^a^
SMM (kg)	25.58 ± 3.18	25.26 ± 3.45	25.53 ± 3.52	0.75	0.74 ^a^
**Blood pressure**					
SBP (mmHg)	110.71 ± 11.90	111.44 ± 15.30	111.95 ± 13.91	0.79	0.81
DBP (mmHg)	77.42 ± 9.63	77.81 ± 10.11	77.82 ± 9.17	0.94	0.72
**Blood parameters**					
FBS (mg/Dl)	87.21 ± 9.14	85.71 ± 7.68	88.36 ± 11.30	0.26	0.64
Total cholesterol (g/dl)	186.93 ± 34.22	185.36 ± 38.54	179.62 ± 34.19	0.35	0.32
TG (mg/dl)	122.21 ± 67.58	109.23 ± 51.23	127.14 ± 79.19	0.28	0.29
HDL (mg/dl)	46.92 ± 9.86	48.41 ± 12.32	44.60 ± 10.01	0.08	0.18
LDL (mg/dl)	95.92 ± 22.08	97.91 ± 25.29	90.78 ± 24.33	0.15	0.19
hs-CRP (mg/L)	4.63 ± 3.97	4.70 ± 4.18	5.78 ± 5.17	**0.06**	**0.05**
HOMA index	3.38 ± 1.16	3.12 ± 1.12	3.48 ± 1.45	0.22	0.38
Insulin (mIU/ ml)	1.20 ± 0.23	1.21 ± 0.22	1.23 ± 0.24	0.63	0.64
MCP-1 (ng/ml)	47.86 ± 90.28	49.30 ± 85.48	54.51 ± 101.29	0.89	0.69
Galectin3 (ng/ml)	3.44 ± 6.91	5.53 ± 7.90	3.99 ± 7.51	0.63	0.48
PAI-1 (ng/ml)	17.10 ± 35.99	11.55 ± 18.08	18.41 ± 29.51	0.49	0.54

SD: Standard deviation; GRS: Genetic risk score; BMI: Body mass index; WC: waist circumference; WHR: waist height ratio; BFM: body fat mass; BF: body fat; BMR: Basal metabolic rate; BMC: bone mineral content; SMM: skeletal muscle mass; SBP: Systolic blood pressure; DBP: Diastolic Blood Pressure; FBS: fasting blood sugar; TG: Triglyceride; LDL: Low density lipoprotein; HDL: High density lipoprotein; hs-CRP: High-sensitivity C-reactive protein; MCP-1: monocyte chemoattractant protein; PAI-1: Plasminogen Activator Inhibitor 1

† Calculated by analysis of variance (ANOVA)

a BMI considered as collinear and this variable adjusted for age, physical activity, and smoking

b Adjusted for age, BMI, physical activity

### Association of the GRS with obesityrelated anthropometric parameters

**Fig. 2:**
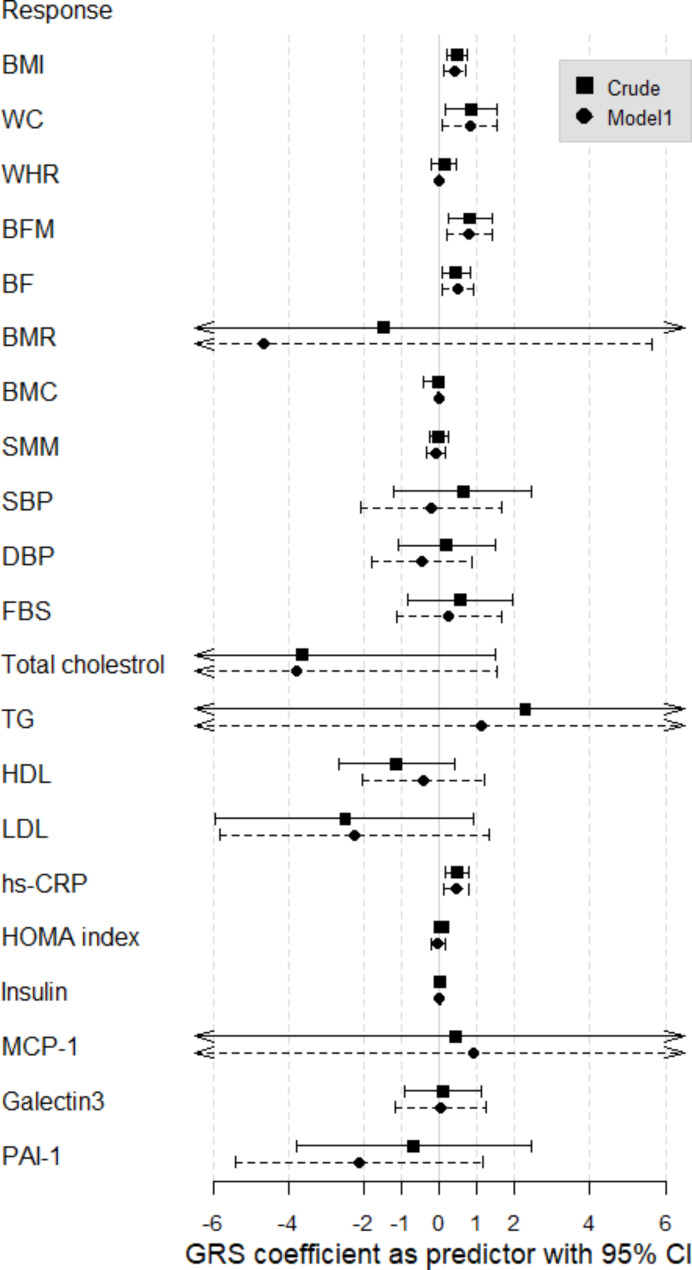
Forest plot for linear regression coefficients, the associations between GRS and the cardiometabolic variables. BMI: Body mass index; WC: waist circumference; WHR: waist height ratio; BFM: body fat mass; BF: body fat; BMR: Basal metabolic rate; BMC: bone mineral content; SMM: skeletal muscle mass; SBP: Systolic blood pressure; DBP: Diastolic Blood Pressure; FBS: fasting blood sugar; TG: Triglyceride; LDL: Low density lipoprotein; HDL: High density lipoprotein; hs-CRP: High-sensitivity C-reactive protein; MCP-1: monocyte chemoattractant protein; PAI-1: Plasminogen Activator Inhibitor 1

The crude model revealed a significant relationship between the GRS and some anthropometric variables, including BMI (β = 0. 49, 95%CI = 0.22 to 0.76, p = 0.001), WC (β = 0. 86, 95%CI = 0.18 to 1.54, p = 0.01), body fat mass (β = 0. 82, 95%CI = 0.25 to 1.39, p = 0.005), and %BF (β = 0. 44, 95%CI = 0.06 to 0.82, p = 0.02) (Table 3) (Fig. 2).

**Table 3:** Association of GRS on cardiometabolic risk factors among obese and overweight female subjects

Variables	GRS						
	**Crude**			**Model1**			
	**Β**	**95 CI**	**P-value**	**Β**	**95 CI**	**P-value**	**R2**
BMI (Kg/m^2^)	0.49	0.22 to 0.76	**< 0.001**	0.42	0.13 to 0.71	**0.004**	
**Body composition**							
WC (cm)	0.86	0.18 to 1.54	**0.01**	0.81	0.08 to 1.53	**0.02** ^a^	0.01
WHR (ratio)	0.12	-0.20 to 0.45	0.46	0.004	0.00 to 0.008	**0.07** ^a^	0.01
BFM (kg)	0.82	0.25 to 1.39	**0.005**	0.79	0.19 to 1.39	**0.01** ^a^	0.01
BF (%)	0.44	0.06 to 0.82	**0.02**	0.49	0.08 to 0.89	**0.01** ^a^	0.01
BMR (kcal)	-1.49	-10.95 to 7.96	0.75	-4.66	-14.99 to 5.66	0.37 ^a^	0.006
BMC (g)	-0.02	-0.44 to 0.005	0.12	-0.02	-0.05 to -0.001	**0.04** ^a^	0.02
SMM (kg)	-0.01	-0.25 to 0.23	0.92	-0.09	-0.35 to 0.16	0.46 ^a^	0.08
**Blood pressure**							
SBP (mmHg)	0.62	-1.20 to 2.44	0.50	-0.21	-2.10 to 1.67	0.82	0.081
DBP (mmHg)	0.20	-1.09 to 1.50	0.75	-0.47	-1.81 to 0.86	0.48	0.071
**Blood parameters**							
FBS (mg/Dl)	0.54	-0.85 to 1.95	0.44	0.26	-1.13 to 1.66	0.71	0.10
Total cholesterol (g/dl)	-3.63	-8.74 to 1.47	0.16	-3.78	-9.09 to 1.53	0.16	0.09
TG (mg/dl)	2.28	-7.68 to 12.25	0.65	1.12	-9.65 to 11.90	0.83	0.07
HDL (mg/dl)	-1.13	-2.66 to 0.40	0.14	-0.42	-2.05 to 1.21	0.61	0.008
LDL (mg/dl)	-2.5	-5.95 to 0.92	0.15	-2.24	-5.81 to 1.31	0.21	0.084
hs-CRP (mg/L)	0.46	0.14 to 0.78	**0.005**	0.44	0.11 to 0.78	**0.009**	0.15
HOMA index	0.04	-0.13 to 0.23	0.61	-0.03	-0.22 to 0.16	0.76	0.10
Insulin (mIU/ ml)	0.01	-0.01 to 0.05	0.33	0.01	-0.02 to 0.04	0.45	0.07
MCP-1 (ng/ml)	0.43	-8.21 to 9.07	0.92	0.91	-8.00 to 9.83	0.84	0.009
Galectin3 (ng/ml)	0.09	-0.93 to 1.12	0.85	0.04	-1.17 to 1.26	0.93	0.03
PAI-1 (ng/ml)	-0.68	-3.80 to 2.43	0.66	-2.13	-5.41 to 1.15	0.20	0.06

SD: Standard deviation; R2: R-squared; GRS: Genetic risk score; BMI: Body mass index; WC: waist circumference; WHR: waist height ratio; BFM: body fat mass; BF: body fat; BMR: Basal metabolic rate; BMC: bone mineral content; SMM: skeletal muscle mass; SBP: Systolic blood pressure; DBP: Diastolic Blood Pressure; FBS: fasting blood sugar; TG: Triglyceride; LDL: Low density lipoprotein; HDL: High density lipoprotein; hs-CRP: High-sensitivity C-reactive protein; MCP-1: monocyte chemoattractant protein; PAI-1: Plasminogen Activator Inhibitor 1

† Calculated by linear regression

Model1: Adjusted for age, BMI, physical activity, and energy intake

a BMI considered as collinear and this variable adjusted for age, physical activity, and smoking

After adjustment for confounding factors (age and physical activity), a significant positive association was observed between BMI (p = 0.004), WC (p = 0.02), body fat mass (p = 0.01), and %BF (p = 0.01) and GRS. Also, we observed a significant negative association of the GRS with BMC (β= -0.02, 95%CI= -0.05 to -0.001, p = 0.04). Moreover, a marginally significant positive association was seen between WHR (β = 0. 004, 95%CI = 0.00 to 0.008, p = 0.07) and GRS in adjusted model.

### Association of the GRS with obesity-related metabolic traits

The findings revealed a significant positive relationship between hs-CRP (= 0.46, 95% CI = 0.14 to 0.78, p = 0.005) and GRS. But no significant association of the GRS with other obesity-related metabolic variables such as SBP, DBP, FBS, TG, TC, LDL-C, VLDL-C, HDL, HOMA-IR, insulin, MCP-1, PAI-1, and Galectin3 was seen in the crude model (Table 3) (Fig. 2). After controlling for confounder factors such as age, physical activity, and BMI, the significant positive association between hs-CRP (p = 0.009) and GRS was maintained. But other variables did not show any significant association with GRS among obese and overweight women.

## Discussion

This cross-sectional study investigated the relationship of genetic risk score by obesity-related genetic markers, including MC4R (rs17782313), CAV-1 (rs3807992), and Cry-1 (rs2287161), with cardiometabolic risk factors in overweight and obese women. There are no studies focusing on obesity-GRSs with overweight and obesity in Iranian overweight and obese populations, which reinforces the potential of our GRS analysis. Our results showed that women in T3 of GRS had a significantly higher BMI and percentage of body fat. Also, a borderline significant difference across genetic risk scores was observed for body fat mass and hs-CRP. A significant positive association was observed between BMI, WC, body fat mass, %BF, hs-CRP, and GRS. Additionally, the GRS was negatively correlated with the BMC. WHR and GRS also showed a marginally significant positive correlation. However, there were no significant associations between other variables and GRS among obese and overweight women.

We have shown that higher GRS has been associated with higher BMI, which in turn, higher BMI is a risk factor for cardiometabolic disease and might be a useful tool in identifying Iranian women at high risk for obesity and cardiometabolic disease. According to Aslumi et al., the GRS based on 15 gene variants with cardiometabolic traits is associated with a higher BMI and also significantly increases WC and triglycerides through the presence of a low-protein diet [47]. Moreover, one cross-sectional study showed that GRS for adult BMI was associated with childhood and adolescent weight gain in an African population. These findings suggest that genetic susceptibility to higher adult BMI can be detected in childhood in this African population, and preventing adult obesity should begin at an early age, according to these findings [48]. Several SNPs have been associated with obesity-related traits in cross-sectional studies [47, 49]. One cross-sectional study showed the combined effect of several genetic variants on obesity in Pakistanis and indicated the prediction of anthropometric traits by a GRS for obesity in a sample of Pakistanis [50], and several studies show the positive relationship between obesity indices such as BMI, BF%, BFM, WC, and WHR with cardiometabolic diseases [51–53]. The obesity-GRS was also shown to be significantly linked to higher body fat mass among Finnish children and adolescents [54]. Previous studies showed that the MC4R variant has an association with higher BMI and WC in children and adults [55, 56]. A-allele carriers of the CAV-1, which is abundant in adipocytes [25], had a significantly higher BMI [29, 30]. Also, Cr*y* − 1 has an important role in metabolism [31] and the C-allele of the Cry-1 polymorphism can lead to significantly higher obesity indices such as BMI, weight, WC, and hip circumferences [34]. Moreover, we found a significant positive association between WC and GRS, and the association between central obesity and mortality risk was reported in a recent study of 42,702 participants in Europe [57]. This has particular significance for Asian populations, whose BMIs are within the normal range but are associated with increased visceral adiposity [47, 51].

In line with our study, several studies show a positive relationship between inflammatory markers such as hs-CRP and GRS. One study showed inflammatory markers such as MCP-1 are higher in risk-allele carriers of CAV1 with unhealthy diet patterns [58]. One study suggests that CAV-1 inhibits the activity of the endothelial nitric oxide synthase (eNOS) and HDL receptor in caveolae [58]. Numerous genetic loci have been identified that affect serum CRP levels, including the CRP gene itself, the APOE gene, and the IL6R gene, and CRP levels were higher among participants with a higher GRS [59]. An analysis of the effect of GRS on cardiovascular disease revealed significant mediation by established cardiovascular risk factors [60]. In the Framingham Heart Study, a GRS composed of 13 SNPs associated with CVD was an independent predictor of cardiovascular events and offers modest improvements in reclassification for incident cardiovascular events [61]. In the Diabetes Heart Study, GRSs derived from 13 to 30 SNPs have been shown to be associated with prevalent CVD, and these associations have been extended to include all-cause and CVD mortality [62].

Also, we observed a significant negative association between the GRS and BMC. Other studies found that the rs17782313 C risk allele of MC4R has an inverse association with BMC. It can be related to lower fat-free mass in people with the MC4R risk allele [63]. Because FFM has an important role in bone mineral density. Also, it has been shown that the MC4R genotype, which is an independent determinant of fat mass [64], is also related to bone mass in children [65]. One study suggested a relationship between CAV1 and bone health and metabolism [66]. They saw knockdown of *Cav-1* in animal studies led to a decrease in osteoclast differentiation, osteoclast genesis, and bone homeostasis, and so Cav-1 had a positive role in osteoclast differentiation of female but not male mice, and it can be associated with estrogen-induced signaling processes and the protective effect of estrogen on osteoclasts [66]. However, we observed a significant negative association between the GRS and the BMC. This may be because we assess three distinct genes and not the effects of each one separately. Each mammalian cell possesses its own molecular circadian clock, and it consists of a unit of positive (CLOCK and BMAL1) and negative elements (CRY, PER, and REV-ERB alpha), that numerous clock genes are controlled by molecular clocks [66]. The bone volume was increased in *Cry* deficient mice [67]. Indeed, there is an association between circadian rhythms and osteoblast differentiation, and mineral deposition genes are predominantly associated with circadian rhythm patterns [67, 68].

To the best of our knowledge, this study is the first to examine the association of GRS and cardiometabolic risk factors among overweight and obese women. Therefore, the use of GRS is a more effective method of estimating the genetic risk of overweight/obesity when comparing different multiple common risk variants than individual risk variants, especially when the sample size is not very large. A strength of the present study is that it was performed in a developing country, where information about diet-disease associations is limited. However, several limitations should be considered. As a result of the cross-sectional design of the study, it was impossible to draw any causal linkage. Another limitation is that the current study had a small sample size and was conducted on a population from Tehran, and therefore the results might not be generalizable to the other cities in Iran.

## Conclusion

In conclusion, we found a positive significant association between GRS and some cardiometabolic risk factors in overweight and obese women. However, as a result of the limited available literature performed in this regard, where most have been conducted with Asian participants, further prospective studies in different populations are needed to confirm and compare the veracity of our findings.

## Data Availability

The datasets used and/or analyzed during the current study are available from the Khadijeh Mirzaei on reasonable request.
